# Serum Hyaluronic Acid: A Promising Marker of Hepatic Fibrosis in Chronic Hepatitis B

**DOI:** 10.4103/1319-3767.43272

**Published:** 2008-10

**Authors:** Gamal Shiha

**Affiliations:** Department of Internal Medicine, Gastroenterology and Liver Unit, Almansoura Faculty of Medicine, Almansoura, Egypt. E-mail: g_shiha@hotmail.com

The chronic hepatitis B (CHB) virus affects 350 million individuals worldwide. The infection that is caused by the virus is accompanied by a progressive deposit of hepatic fibrosis, which in turn may lead to cirrhosis.[1,2] Liver biopsy is the gold standard for the assessment of the fibrosis, but it is hampered by several disadvantages such as poor patient compliance, sampling error, and poor intra- and inter-observation concordance.[3] Noninvasive evaluation of liver fibrosis is thus a subject of great clinical interest.

Many parameters for noninvasive diagnosis of liver fibrosis were studied extensively in the past.[4–8] These parameters include routine laboratory tests, serum markers of fibrosis and inflammation, ultrasonography and radiological imaging studies.[9] However, at present, none of these tests or markers alone is accurate or reliable in predicting hepatic fibrosis, either in CHB or other related diseases like chronic hepatitis C (CHC).

Ideally, a marker of hepatic fibrosis should be liver specific. It should also be able to measure the activity of the matrix deposition, reflect the underlying fibrosis, irrespective of the cause, should be easy to perform and yet sensitive enough to distinguish between the different stages of fibrosis.

In the liver, hyaluronic acid (HA) is mostly synthesized by the hepatic stellate cells and degraded by the sinusoidal endothelial cells.[10] It has been shown that serum HA levels increase in chronic liver diseases and that progressive liver damage can be identified early by serum HA assessment.[11,12] In patients with chronic liver diseases of various etiologies, increases in serum HA levels occur together with the development of liver fibrosis. In alcoholic liver disease, Tran ***et al.***, found that HA was the best marker for the prediction of severe fibrosis, where HA had a diagnostic accuracy of 91.1%.[13] Prior to that, Oberti ***et al.*** had observed that HA and prothrombin index were the best predictive factors.[14] There are many studies about serum HA as a fibrosis marker in CHC.[9,15–18] One of these studies has suggested cut-off values of HA to discriminate the different stages of liver fibrosis, but it was limited by the number of cirrhotic patients included amounting to only 5% of the study group.[17] In literature, there are few studies about the noninvasive diagnosis of fibrosis in CHB.[3,19–21] The results obtained in patients with CHB appear relevant since HCV and HBV infection may differ significantly in terms of fibrosis progression and the presence of related markers.[22,23] This was elegantly demonstrated in a recent study that enrolled both CHB and CHC patients who underwent liver biopsy and had FibroScan performed on the same day. The study concluded that the efficacy of liver stiffness as measured by FibroScan was superior in detecting fibrosis in patients with CHC than in patients with CHB.[24] Therefore, it appears that data pertaining to CHC cannot be routinely applied to CHB.

This adds to the importance of the work done by Geramizadeh ***et al.***, in this issue of the Saudi Journal of Gastroenterology, who tried to validate the value of HA as a simple laboratory test to discriminate between patients with and without significant fibrosis in CHB.[25] This study included 93 patients with CHB, who had liver biopsy. Interestingly, any biopsy specimen of less than 2 cm was excluded, which is a very important factor in increasing the accuracy of liver biopsy reports. Also, examining liver pathology specimens by two independent experts, blinded to the clinical and laboratory data, certainly minimized the interobserver differences. This is crucial in studies evaluating noninvasive fibrosis markers, wherein the ‘gold standard’ of liver biopsy should ideally find a replacement in real ‘gold’. This was not the case in similar studies,[19] where the biopsy size was 10 mm only and all biopsies were reviewed by a single pathologist whose experience was not reported.

In the study of Geramizadeh ***et al.***, a cut-off value of HA 113 ng/ml was chosen for identifying the absence or presence of mild fibrosis and another value of HA 181.9 ng/ml was chosen for identifying the absence or presence of severe fibrosis and cirrhosis. This is different from the study of Montazeri ***et al.***, where a cut-off value of 126.4 ng/ml could discriminate mild fibrosis from extensive fibrosis.[19] It should be noted that in both the studies, the number of patients with severe fibrosis and cirrhosis was small (16 and 17%, respectively) and there was no distinction between incomplete and established cirrhosis, which is potentially important in clinical practice in relation to decision making for treatment and prognosis issues.

Moreover, the present study lacked data about alanine transaminase (ALT) and aspartate transaminase (AST) activities, HBV DNA level and HBeAg status. This is an important limitation, as these biochemical and virological parameters may affect the stage of fibrosis in CHB. This was previously demonstrated in the study by Zeng ***et al.***, where their model of noninvasive diagnosis of liver fibrosis in CHB was applied only to patients with HBeAg positive status and elevated transaminases activity, and was not applied to patients with negative HBV DNA, normal or minimally elevated ALT activity, or HBeAg negative patients.[26] Therefore, future studies concerning the evaluation of HA or other noninvasive markers for the diagnosis of fibrosis of CHB should routinely include these biochemical and virological data. This will not only allow for proper interpretation of fibrosis markers in different phases of CHB, but may also help in comparison of the results of studies including different CHB populations. Besides, a large cohort of cirrhotic patients is needed for the proper evaluation of HA and other noninvasive markers in the diagnosis of cirrhosis. Further large scale studies may allow a more precise cut-off value of HA between each stage of fibrosis, rather than a mere distinction between mild and severe fibrosis. Finally, combining HA level with other serum markers or Fibroscan for assessing hepatic fibrosis should be considered.

In conclusion, this study shows that serum HA is a promising marker of hepatic fibrosis in CHB, but it should be validated in well-designed controlled studies that avoid the limitations of the previous work and consider combination with other simple non-invasive markers of liver fibrosis.
